# Medical Society of the County of Albany, Semi-Monthly Meetings

**Published:** 1873-04

**Authors:** F. C. Curtis

**Affiliations:** Secretary


ART. II.—*Medical Society of the County of Albany.—Semi-Monthly Meeting, March 12th*, 1873.

Reported by F. C. CURTIS, M. D., Secretary.

The name of Dr. G. A. Jones was proposed for membership and referred to the board of censors.

Dr. S. H. FREEMAN read a paper on *Laryngitis*. This, he said, is not the slight inflammation or congestion of the mucous surface of the larynx which is the common attendant upon a simple cold, but an inflammation involving also the sub-mucous tissue, causing engorgement and serous effusion. It is happily of rare occurrence for it is one of the most distressing diseases incident to humanity, and generally proves fatal. Dyspnœa and dysphagia are produced, the patient generally dying of asphyxia unless relieved by active medication or tracheotomy. Cases ending fatally in seven hours from the onset are recorded. It is a disease of robust, adult life almost exclusively.

The treatment of a case recently occurring in his practice had been, first the application of leeches and fomentations to the throat, together with the administration of slightly nauseating doses of antimony and lobelia. Then cantharidal collodion was applied freely over the region of the larynx, all of which seemed to prepare the way for that which gave the patient the greatest relief, viz. the careful and persistent inhalation of the vapor of boiling water.

Dr. FOWLER approved of the use of steam in these cases. He applied it by filling the room with it by means of kettles of boiling water.

Dr. STEVENS said, that having occasion recently to make local application of steam to an ear, he had done so by conducting the steam through a rubber tube, from a vessel of hot water to a common syringe, by means of which it was directed into the ear.

Dr. CURTIS reported, in connection with the subject, a case in which hot steam was accidentally inhaled from a teakettle by a child, producing œdema and inflammation of the air-passages. At the time of the injury little harm seemed to have been done, the child being easily pacified. But three or four hours after breathing began to be difficult, the voice hoarse and febrile action set in.

When seen about five hours after the inhalation breathing was labored and loud, there was aphonia, the skin was suffused, with rapid pulse and hot surface. Ædema of the glottis could be readily distinguished, two hard tumors as large as the end of the finger being felt below the epiglottis. In such a condition tracheotomy or scarifying the œdematous points suggest themselves. In this case no operative interference was proposed, on account of the probable extent of the injury and the low condition of the child. The treatment directed was the application of hot stupes to the throat and chest, and filling the room with the vapor of warm water. She died about 24 hours after the injury, more apparently from depression of the system, or shock, than from dyspnoea.

There was presented also a tube of hard rubber, with a proper curve for passing into the trachea. This was seen to meet with success in the hands of Dr. Wiederhofer at the Children's Hospital in Vienna, in cases of true croup. After being passed through the rima glottidis the breathing became more easy; and more—by passing a feather, trimmed nearly to its tip, through the tube, considerable false membrane was entangled and removed.

Dr. Fowler reported a case of Gangreen of the leg occurring in the course of a hemiplegia. The subject had taken opium to excess for ten years, and was also a habitual drinker. In December 1872 he was paralized in the left side. Nothing unusual occurred until January 10th 1873, when the left foot began to swell, and on the 28th gangreen appeared, spreading gradually until the whole foot and lower part of the leg was involved. His general condition was good until February 18th, when convulsions occurred and two days later he died.

Dr. McNaughton mentioned a case in which gangreen followed paralysis of the right arm, in the case of a man who had always been healthy excepting a sub-acute rheumatism, the heart being sound. Paralysis of the limb came on suddenly without apparent cause, following severe pain of the part, and gangreen occurred in a few days. It proceeded rapidly up the arm until a line of demarkation formed above the elbow. The arm was amputated and the patient recovered. It was supposed that destruction of vitality was due to paralysis. It was noted at the operation that blood flowed from cut arteries in a steady stream as from a vein.

Dr. STEVENS expressed the opinion that gangreen in this case might have been produced by the supply of blood being cut off on account of calcareous degeneration of the arteries.

Dr. J. P. BOYD, Jr., made a report of the autopsy of the late Dr. U. G. Bigelow. Various organs were found in a condition of disease, the most peculiar being a marked diminution in calibre of the intestines in sections. The duodenum was constricted and adherent to the pancreas; the ileum was small throughout, and the descending colon was also reduced in size to near the sigmoid flexure where it was dilated. He thought these contractions of the intestines could be accounted for by hypertrophy of the cellular tissue which was found microscopically; there were no marks of constrictions, and no ulcerations anywhere.

During life Dr. Bigelow suffered much from pain in the abdomen and had been troubled with disturbance of the alimentary canal. He had always been in delicate health, from the time of his entering the profession.

Dr. VAN DERVEER said that he had seen a similar condition in one case only. This person had been troubled for years with obstinate constipation, to relieve which he used enemata habitually. On the morning of the day he died, the enema had been followed by pain which anodynes did not relieve. Post mortem examination showed the intestines, especially the colon, to be of very small calibre in sections, but with no bands of constriction. The cause of death was rupture of the heart.

A report by Dr. STONEHOUSE of "five cases in which the hæmostatic properties of ergotine were tested" was read by the secretary. The drug was administered hypodermically, eight drops being used of a solution of half a drachm to a drachm of water. Two of these were cases of hæmoptisis, one of metrorrhoea, one was renal and one puerperal. In all excepting the last bleeding ceased on its exhibition.

Dr. CRAIG said that this use of the drug was discussed at the late meeting of the State Society and was not spoken favorably of, as in the experience of some severe abscesses had followed its exhibition.

Dr. Boyd said that he has seen it used at Breslau three times a day, in a case of uterine fibroid and no abscess had formed.

---

Semi-Monthly Meeting, March 26th, 1873.

The Society met at the city building at the usual hour. Dr. Van Derveer, President in the chair.

Dr. C. S. Hoyt, Secretary of the Board of Public Charities, addressed the Society on the subject of the care of the Insane.

As to the number of insane people in the country, the census report has not been reliable, which has been shown by the census of insane taken in two or three instances by boards of public charities. It was found in 1871, by Dr. Hoyt, that the total number of insane in this State was 6,775. Of these 1,582 were in the custody of friends, 1,093 in State institutions, 312 in private institutions, 3,552 in city and county poor houses, and 161 in institutions in other States.

In Albany county the number of insane is 223, or 1 to 551 inhabitants, a larger proportion than any other county except Oneida, New York and Kings. Of these, 66 are in the county poor house and 105 in State institutions, the rest being scattered in private institutions, or to other States, or retained in the custody of friends. It has been a matter of interest to find how many have become insane during the year. There have been 761 recoveries and 502 deaths among the insane of the State. By deduction from the total number of insane, it is found that 1,678 new cases of insanity have occurred, or 1 to every 2,612 population.

The circumstances under which the insane have recovered is largely in favor of hospital treatment. Of 761 recoveries, only 43 were of those under the care of friends. It was urged on physicians to send insane patients as soon as possible to the asylum, and also to try and remove the prejudice existing against these institutions, which is largely due to lack of information. The charges that have been made from time to time are, for the most part, unfounded. Those against Bloomingdale are shown to have been entirely malicious. The hope of recovery diminishes as time advances, a large proportion of recoveries being within a year.

It was urged that this county could take care of its insane best by building an asylum of its own, and that it would be more economical. It costs from $20 to $35 to transport patients to distant institutions, which sums would pay the interest on the cost of building. There are now 66 insane in the county poor house because of lack of accommodation elsewhere, and they ought not to be deprived of advantages which the others enjoy; epileptics, too, are a dangerous class to have at large, and for them there is no public provision. He thought that the cost of maintaining 250 patients would be about $3 a week, in an institution having a large vegetable farm attached. This is what is paid by the county for patients at the Willard asylum.

Mr. Hoxsie, overseer of the poor, said that we have 102 patients in the Willard asylum, and 50 or 60 whom he would be glad to send there if there was room. A number of supposed hopelessly insane sent to Willard asylum, when first established, had been returned cured. He spoke highly of the care given there. The Supervisors of this county decided not to build an asylum on account of the expense, but they had appropriated $35,000 for this year's care, the largest sum ever set apart. We shall never have a less number, and it would not take many sums of this amount to erect a home asylum. It might, too, be made renumerative by taking from other counties. The accomodations in the State asylums are entirely insufficient, as shown by the fact that they are crowded, and many patients are now in other States, or in the custody of friends.

Dr. James S. Bailey reported a case of poisoning by aniline. A workman in the employ of the Aniline Company undertook, contrary to the advice of the foreman, to open an iron cask containing aniline oil and arsenious acid. Gas, formed by the intense summer heat, was liberated, and being inhaled caused asphyxia. He was carried home in an insensible condition, which continued for fourteen hours. When seen he had a slow pulse, dilated pupils and cool surface. He was prostrated for two weeks by a bronchial affection, caused by the irritation which the gas produced in the air passages, but made a good recovery. In 1868 Professor Wirtz of Paris, while demonstrating the properties of aniline as a coloring matter to a class, having inhaled the fumes, fell senseless and immediately expired.

Dr. Bailey also reported the history and post mortem of a case of rupture of the œsophagus near the stomach, causing death in twenty-four hours, substances swallowed passing into the cavity of the chest. The accident was probably due to a violent fit of vomiting. The lesion in a sound œsophagus is a rare one. Oppolzer reports having seen but one case.

Dr. Van Derveer presented a case of Pyelitis. The history was brief as the patient had been seen only once during life. He was 45 years of age and a sailor by occupation, having been exposed to great hardships from boyhood. He had been treated for six months at the different dispensaries in the city. The symptoms presented were nausea, vomiting, pain in the lumbar region, difficulty in urinating, with a frequent desire to do so night and day, though but little was passed at a time. Respiration was frequent and labored. The urine had a thick whitish appearance and on examination was found to contain one third albumen and pus in abundance.

*Post mortem.* The body was much emaciated. There were strong pleuritic adhesions over the entire surface of both lungs, evidently of long standing. The lung tissue was healthy. Heart normal.

The liver was somewhat enlarged, pale, and having a fatty appearance. The peritoneal coat when torn up brings along a portion of the substance of the gland, giving the surface of the liver a course, granular look. It was friable. Gall bladder full and the opening of the ductus communis choledochus into the duodenum well marked and distinct.

The Spleen, pancreas and alimentary canal were normal.

Kidneys: The right was firmly held in the lumbar region by adhesive inflammation of the surrounding cellular tissue, and it was removed with difficulty. It was large and fluctuated distinctly. It weighed 12 ounces. The capsule was firmly adherent. The ureter was very much enlarged and thickened. When cut across near the bladder a purulent fluid escaped. On laying the kidney open pus flowed freely from the medullary portion. The tubular portion was completely occupied by from 12 to 15 distinct abscesses, and these seem to have destroyed particularly the pyramids, occupying their places. There was a well defined wall existing between the most of the abscesses, all of them having their exit toward the pelvis of the kidney. There was not a well defined pyramid left and but a small portion of the cortical substance.

The left kidney was easily removed. It was large and of a pale rose color. It weighed 7 ounces, being 6¼ inches in length, 3½ in width and 2 in thickness. The capsule was easily removed, the surface under it being coarse and granular. Laying open the kidney the pyramids were well defined, but the organ on section was coarse and granular. In consistence it was soft and paler than normal. Microscopical examination of it, by Dr. Blatner, showed a diffuse, cellular hyperplasia of the connective tissue near the convoluted urinary tubuli and the malpighian corpuscles. There were numberless young cells and some oil globules perceptible. The malpighian corpuscles were also somewhat shriveled.

